# Automated fluorescent miscroscopic image analysis of PTBP1 expression in glioma

**DOI:** 10.1371/journal.pone.0170991

**Published:** 2017-03-10

**Authors:** Behiye Kaya, Evgin Goceri, Aline Becker, Brad Elder, Vinay Puduvalli, Jessica Winter, Metin Gurcan, José Javier Otero

**Affiliations:** 1 Department of Pathology, Division of Neuropathology, The Ohio State University College of Medicine, Columbus, Ohio, United States of America; 2 Akdeniz University, Engineering Faculty, Computer Engineering Department, Antalya, Turkey; 3 Department of Radiation Oncology, The Ohio State University, Columbus, Ohio, United States of America; 4 Department of Neurological Surgery, The Ohio State University, Columbus, Ohio, United States of America; 5 Division of Neuro-oncology, The Ohio State University Wexner Medical Center, Columbus, Ohio, United States of America; 6 Department of Biomedical Engineering, The Ohio State University, Columbus, Ohio, United States of America; 7 William G. Lowie Department of Chemical and Biomolecular Engineering, The Ohio State University, Columbus, Ohio, United States of America; 8 Department of Biomedical Informatics, The Ohio State University, Columbus, Ohio, United States of America; Thomas Jefferson University, UNITED STATES

## Abstract

Multiplexed immunofluorescent testing has not entered into diagnostic neuropathology due to the presence of several technical barriers, amongst which includes autofluorescence. This study presents the implementation of a methodology capable of overcoming the visual challenges of fluorescent microscopy for diagnostic neuropathology by using automated digital image analysis, with long term goal of providing unbiased quantitative analyses of multiplexed biomarkers for solid tissue neuropathology. In this study, we validated PTBP1, a putative biomarker for glioma, and tested the extent to which immunofluorescent microscopy combined with automated and unbiased image analysis would permit the utility of PTBP1 as a biomarker to distinguish diagnostically challenging surgical biopsies. As a paradigm, we utilized second resections from patients diagnosed either with reactive brain changes (pseudoprogression) and recurrent glioblastoma (true progression). Our image analysis workflow was capable of removing background autofluorescence and permitted quantification of DAPI-PTBP1 positive cells. PTBP1-positive nuclei, and the mean intensity value of PTBP1 signal in cells. Traditional pathological interpretation was unable to distinguish between groups due to unacceptably high discordance rates amongst expert neuropathologists. Our data demonstrated that recurrent glioblastoma showed more DAPI-PTBP1 positive cells and a higher mean intensity value of PTBP1 signal compared to resections from second surgeries that showed only reactive gliosis. Our work demonstrates the potential of utilizing automated image analysis to overcome the challenges of implementing fluorescent microscopy in diagnostic neuropathology.

## Introduction

Translation of basic scientific findings to improved clinical decision-making for neuro-oncology will require implementing unbiased, multiplexed, and objective histopathology interpretation. The current *status quo* suffers from biased, mainly uniplexed, and subjective histopathology interpretation. To improve the *status quo*, we must overcome multiple technical barriers including tissue processing, image capture, and image analysis. First, inconsistencies in antibody generation and validation represent well-recognized problems in immunohistochemistry [1], and therefore rigorous validation of all antibodies are required. The College of American Pathologists (CAP) has issued guideline policies and recommendations for immunohistochemistry validation in clinical labs [2]. However, these guideline policies do not address issues regarding antibody generation by vendors nor antibody-biomarker interaction validation. With current CAP guidelines, an antibody generated from an unknown epitope (e.g., HELA cell nuclear extract) could be validated as a biomarker for a clinical lab, or a distributor could change production procedures without changing the reagent’s catalogue number and thus not trigger a new validation need in the clinical lab. Unfortunately, multiplexing, routinely performed in hematopathological flow cytometry assays, is fraught with caveats in solid tissue histology. In addition to frequent incompatibilities in antigen retrieval methods between different epitopes, formalin-fixed paraffin embedded (FFPE) brain tissues show significant autofluorescence that complicates visual interpretation of brain biopsies. These challenges include erythrocyte autofluorescence [3], autofluorescence due to FFPE processing [4], and autofluorescence in some brain cells [5]. In addition, epifluorescent image acquisition shows inconsistencies in emitted light intensities between image captures if using halogen bulbs due to the well-documented decrease in bulb-intensity as bulb-age increases, further introducing significant challenges in utilizing fluorescent microscopy in biomarker quantification.

**Fig 1:**
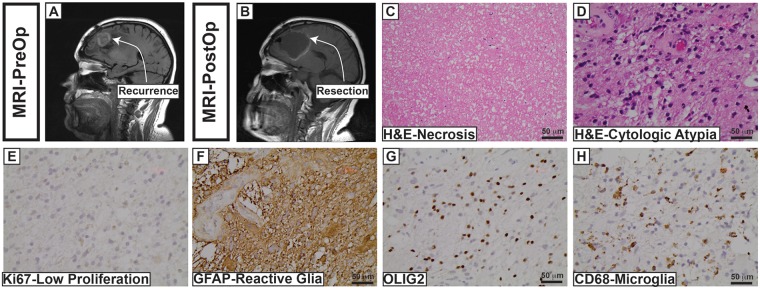
Diagnostic challenges at glioblastoma recurrence. A recurrent frontal lobe lesion highly suspicious for GB (A) is resected (B) and shows significant necrosis (C) with foci of cytologically atypical astrocytes (D). Standard immunohistochemical evaluation of FFPE tissue is shown in E-H. Molecular marker and interpretation are shown in the bottom left of each panel. Interpretation: Abundant necrosis, low ki67 labelling in the background of marked reactive gliosis and inflammatory cells suggested no significant disease progression in the tissue despite the concerning radiographic appearance. Methodology: Note, “standard IHC” refers to DAB 2nd antibody reaction precipitate followed by hematoxylin counterstain as shown in E-H.

We aimed to overcome some of the aforementioned challenges by using glioblastoma (GB) recurrence as an experimental paradigm. GB is associated with dismal survival and is initially detected by radiological imaging and treated with maximum safe resection for debulking and confirmation of diagnosis, followed by standardized chemoradiation therapy (chemoRT) and adjuvant chemotherapy [6]. Identifying progressive areas of gadolinium enhancement in brain raises concern for post-surgical recurrence. However, new areas with gadolinium enhancement after chemoRT may represent true tumor progression or pseudo-progression (a treatment reaction). True tumor progression indicates treatment failure and requires treatment change, whereas pseudo-progression permits conservative management. Distinguishing samples showing predominantly reactive changes versus recurrent glioblastoma represents a major diagnostic challenge in neuropathology and is instrumental in clinical decision-making. For instance, Kim et al. demonstrated that patients in which less than 20% of their specimen showed recurrent tumor had a longer survival probability after their second surgery and an improved overall survival probability compared to patients who underwent a second surgery in which their specimen was composed of >20% recurrent tumor [7]. However, these designations were based solely on morphological parameters, which are challenging in the context of recurrent neoplasms. Fig 1 illustrates an example of such a diagnostic challenge. Therapy-induced cytological atypia is difficult to distinguish between reactive, non-neoplastic cells, and thus biomarkers such as OLIG2, GFAP, and CD45 are typically used to parse out the different cell types in the tissue. Despite utilizing multiple biomarkers in uniplex, the designation of reactive versus recurrent glioblastoma is highly subjective. The goal of this study was to illustrate proper antibody-epitope validation and implement automated image analysis capable of removing confounding background fluorescence in FFPE neuropathology tissue interpretation to test the extent to which a candidate biomarker, Polypyrimidine tract binding protein 1 (PTBP1), could distinguish reactive gliosis versus recurrent glioblastoma.

## Material and methods

### Patient selection

Retrospective cases were evaluated from the Pathology Tissue Archives of The Ohio State University Wexner Medical Center under an IRB approved protocol (2014C0062). (Demographic information Table 1). To develop this technique, we utilized a total of 29 surgical neuropathology cases (4 cases of recurrent glioblastoma, 4 reactive gliosis, 16 pilocytic astrocytoma, 5 de novo glioblastoma). Designations of recurrent glioblastoma or reactive gliosis were determined by a neuropathologist based on histology and biomarker expression (JJO).

**Table 1:** Patient demographics.

Patient Number	Age	Gender	Tumor Location	Diagnosis
Patient#1	34	M	Frontal lobe	Reactive Gliosis
Patient#2	31	F	Left Temporal lobe	Reactive Gliosis
Patient#3	41	M	Left Temporal lobe	Reactive Gliosis
Patient#4	66	M	Right Temporal lobe	Reactive Gliosis
Patient#5	64	F	Right Temporal lobe	Recurrent Glioblastoma
Patient#6	73	F	Left Parietal lobe	Recurrent Glioblastoma
Patient#7	56	M	Right Temporal lobe	Recurrent Glioblastoma
Patient#8	61	M	Left Occipital lobe	Recurrent Glioblastoma
Patient#9	59	M	Left Orbit	Pilocytic Atrocytoma
Patient#10	22	F	Posterior fossa	Pilocytic Atrocytoma
Patient#11	46	M	Frontal lobe, intraventricular region	Pilocytic Atrocytoma
Patient#12	18	M	Right Temporal lobe	Pilocytic Atrocytoma
Patient#13	20	M	Cerebellum	Pilocytic Atrocytoma
Patient#14	20	M	Cerebellum	Pilocytic Atrocytoma
Patient#15	37	M	Right intraventricular region	Pilocytic Atrocytoma
Patient#16	36	F	Cerebellum	Pilocytic Atrocytoma
Patient#17	33	F	Left Cerebellum	Pilocytic Atrocytoma
Patient#18	34	M	Forth Ventricular mass	Pilocytic Atrocytoma
Patient#19	19	F	Cerebellum	Pilocytic Atrocytoma
Patient#20	33	M	Right Temporal lobe	Pilocytic Atrocytoma
Patient#21	58	F	Cerebellum	Pilocytic Atrocytoma
Patient#22	45	F	Left Occipital lobe	Pilocytic Atrocytoma
Patient#23	19	F	Clionidal tumor	Pilocytic Atrocytoma
Patient#24	24	F	Mid brain tumor	Pilocytic Atrocytoma
Patient#25	77	F	Left Occipital lobe	De novo glioblastoma
Patient#26	64	M	Right Frontal lobe	De novo glioblastoma
Patient#27	69	M	Left Temporal lobe	De novo glioblastoma
Patient#28	69	M	Left frontal lobe	De novo glioblastoma
Patient#29	74	M	Left Parietal lobe	De novo glioblastoma

### Immunohistochemistry

All animal procedures were done under supervision of The Ohio State University’s IACUC oversight (protocol number 2012A000000162-R1). All tissues were fixed in phosphate buffered 4% formalin, dehydrated by graded ethanol washes and embedded in wax using routine techniques. All sections were cut at 5 μm thickness and mounted on glass cover slips using routine techniques in clinical histopathology laboratories. Immunohistochemistry with fluorescent detection was performed by blocking sections for 30 min in 5% normal goat serum/PBS, incubation with primary antibody for overnight at 4°C and incubation with anti-mouse IgG and anti-rabbit IgG based secondary antibodies for 1 h at room temperature prior to staining with DAPI and mounting. Heat based antigen retrieval (10 mM Citrate, 99C, 20 min) was performed on all sections to enhance immunodetection. Antibodies were obtained from the following sources and used at the following dilutions and incubation times/temperatures: PTBP1 rabbit monoclonal antibody clone EPR9048 (Millipore, MABE623) 1: 1:250, overnight at 4°C.

### Cell culture knockdown experiments and cell plug assay

Glioblastoma cell line, LN229 (kind gift of Dr. Palanichamy), were grown in DMEM/F12 with 10% FBS. For knockdown experiment, glioblastoma cells were transfected with siRNA by jetPRIME Transfection Reagent (VWR, 89129–922). The siRNA corresponding to PTBP1 mRNA sequence was obtained from Life Technologies (s11434). Twenty-four hours post-transfection, the cells were detached with Accutase (Life Technologies, A11105-01), counted, and reseeded in a 6-well tissue culture dish for a second transfection and incubated overnight. Whole cell protein lysates were extracted after overnight incubation in transfection mix. Thus, the protein lysates represent cells transfected twice with siRNA targeting PTBP1.

### Western blotting

Protein samples were prepared from total cell lysates and separated by 10% SDS-PAGE gel (15 μg per lane), transferred to a nitrocellulose membrane and processed for western blotting analyses with the anti-PTBP1 (Millipore, MABE623), and anti-GAPDH (Millipore, MAB374) antibodies. The results were evaluated using densitometry in FUJI. Primary antibodies were incubated at room temperature for 2 h with the 1:10000 dilution for the PTBP1 rabbit monoclonal antibody clone EPR9048 and 1:1000 dilution for GAPDH mouse monoclonal antibody clone 6C5. Three washes in TBS-T were performed before addition of secondary antibody at 1:10000 dilution for 1 h at room temperature. Three more washes in TBS-T were done before visualization.

### Photomicroscopy

Images for quantitative analysis were obtained from confocal analysis (Zeiss LSM 700), objective lens 20x and captured as a.czi file, opened in FIJI, then colors split and saved as.tiff files. Seven randomized images were captured per specimen and supplied without modifications to the image analysis team for quantification. For epifluorescent quantifications, images were captured with a Zeiss Axioskop2 Mot Plus as.TIF files. Individual nuclei were selected in FIJI and the mean gray value of PTBP1 was measured.

### Statistical analysis

For traditional pathological scoring, representative images were evaluated by experienced neuropathologists (JO and AB) and PTBP1 scores using two systems: (A) a 0–3 point scale showing 0 = no staining, 1 = nuclear staining in <10% of the tumor cells, 2 = nuclear staining in >10% but < 70% of tumor cells, and 3 = nuclear staining in >70% of the tumor cells; (B) a 0–4 point scale showing 0 = no staining, 1 = nuclear staining in 1–25% of the cells, 2 = nuclear staining in 25%-50% of the cells, 3 = nuclear staining in 50%-75% of the cells, and 4 = nuclear staining in >75% of the cells. The scores from both pathologists were averaged for each case and the mean from each group was tested using Anova/Tukey HSD. For concordance/discordance barplots, each case was categorized as showing interobserver concordance or discordance. Concordance was defined as a difference of <1 point score on the 3 or 4 point scale. For statistical hypothesis testing of data obtained from the objective image analysis workflows, data from each image was pooled into each category, and tested by ANOVA/Tukey HSD testing. All statistical hypothesis testing was performed using R(3.2.4 GUI 4.678 Maverick Build). Graphs were also plotted with descriptive statistics in R using boxplot and barplot functions. Mean epifluorescnet signaling for the siRNA knowckdowns was tested using T-test. Cohen’s kappa coefficient was determined by taking all ratings on each tumor class’s image by two experienced neuropathologists (AB and JJO), and was performed in R using the irr library.

### Image analysis

Detailed image analysis techniques are delineated in S1 File and S1–S3 Figs.

## Results and discussion

### Rabbit monoclonal anti-PTBP1 clone EPR9048 antibody specifically binds to PTBP1

**Fig 2:**
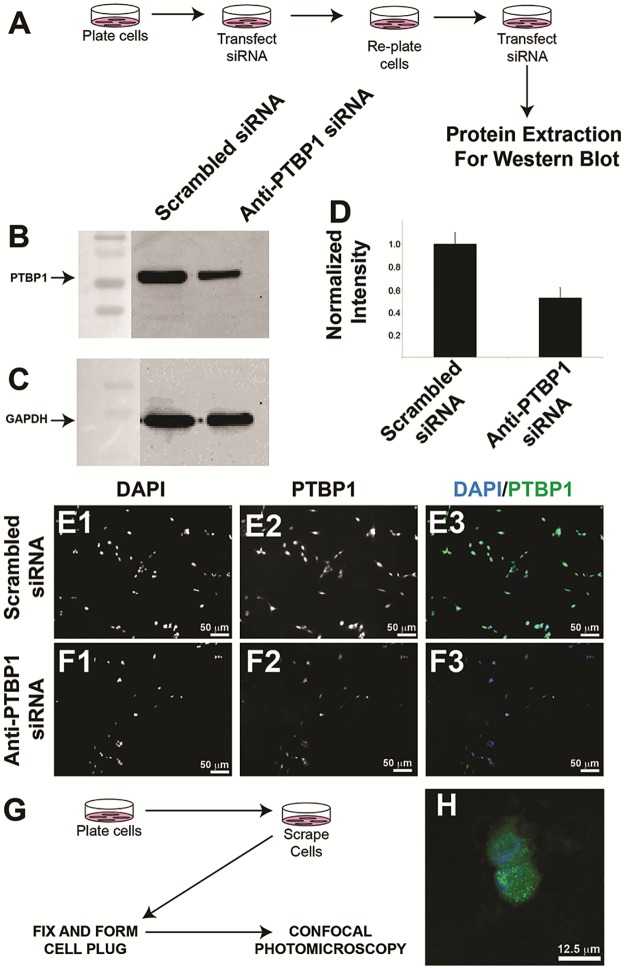
PTBP1 antibody validation. (A) siRNA knock-down approach workflow for PTBP1 knockdown. (B) Western blotting of total cell lysates of the siRNA knockdown cells using anti-PTBP1 antibody. (C) Western blotting of total cell lysates from the siRNA knockdown cells using GAPDH as a loading control. (D) Densitometric analysis of western blotting data pooled from three independent experiments. (E) Immunofluorescent analysis of PTBP1 in scrambled siRNA treated (E1-E3) and anti-PTBP1 siRNA treated (F1-F3). (G) Work flow for cell plug formation to test PTBP1 in glioma. (H) Immunofluorescent stain of PTBP1 in glioma cell line. Cell lines used in B-D were LN229, and cells used for E-H were U251. Mean intensity gray values for PTBP1 knockdown cells were 40.4% decreased relative to scrambled transfected cells (mean PTBP1 density normalized to dapi density for scrambled treated = 0.71 (n = 75 cells), mean PTBP1 density normalized to dapi density for PTBP1 siRNA knockdown = 0.43 (n = 71 cells), p = 2.7 X 10^−12^ by two-tailed homoscedastic T-test.

Polypyrimidine tract binding protein 1 (PTBP1) belongs to the subfamily of ubiquitously expressed heterogeneous nuclear ribonucleoproteins (hnRNPs). Due to its role in RNA processing and nucleolar function, PTBP1 shows predominantly nuclear localization in tissues. Recently, PTBP1 gene amplification and overexpression has been noted in glioblastoma [8]. Specifically, Ferrarese, et al. demonstrated that the alternative splicing of Annexin 7 was mediated by *PTBP1*, and that Annexin 7 interacted with the EGFR signaling pathway by decreasing the endosomal targeting of EGFR, ultimately leading to promotion of the EGFR signaling cascade. Prior reports in the diagnostic pathology literature have also suggested that PTBP1 is elevated in glioblastoma [9, 10]. Multiple PTBP1 antibodies have been utilized in the literature, yet few have been appropriately validated. For example, PTBP1 antibody clone SH54 has been utilized in over 100 citations; yet the immunogen utilized to generate this clone is HELA cell nuclear extract, and the validation of this antibody clone was performed by exogenous expression of PTBP1-GFP cDNA fusion protein [11]. Such lax antibody validation does not meet current publication standards in many biomedical research journals [1], and is particularly worrisome for a gene such as PTBP1, which is expected to have over 15 splice variants (See *Ensembl* gene browser: http://useast.ensembl.org/Homo_sapiens/Gene/Summary?db=core;g=ENSG00000011304;r=19:797075-812327). We therefore set forth to validate anti-PTBP1 clone EPR9048, a monoclonal rabbit antibody generated by immunizing rabbits with human PTBP1 synthetic peptide. To test this, we transfected LN229 cells with siRNA targeting PTBP1. As a control, scrambled siRNA was transfected into LN229 cells. Whole protein lysates were extracted, and resolved by SDS-PAGE. As a loading control, we evaluated GAPDH band intensity. Western blotting analysis demonstrated a significant reduction in LN229 cells transfected with anti-PTBP1 siRNA (Fig 2A-2D). We also validated this antibody by immunofluorescence microscopy of U251 glioma cells fixed on glass cover slips. Here, we subjected U251 cells to similar knock-down parameters for PTBP1 as above, but then fixed the cells with 4% PFA and performed routine immunocytochemistry which demonstrated overall reduced immunofluorescence intensity in the U251 cells (Fig 2E-2F). We also validated this antibody for formalin fixed, paraffin embedded preparations in U251 cell plugs (Fig 2G-2H). We conclude that anti-PTBP1 clone EPR9048 detects PTBP1, and can be utilized for western blotting, immunocytochemistry, and immunohistochemistry of FFPE tissues.

### PTBP1 is differentially expressed in embryonic and post-natal mouse brains

**Fig 3:**
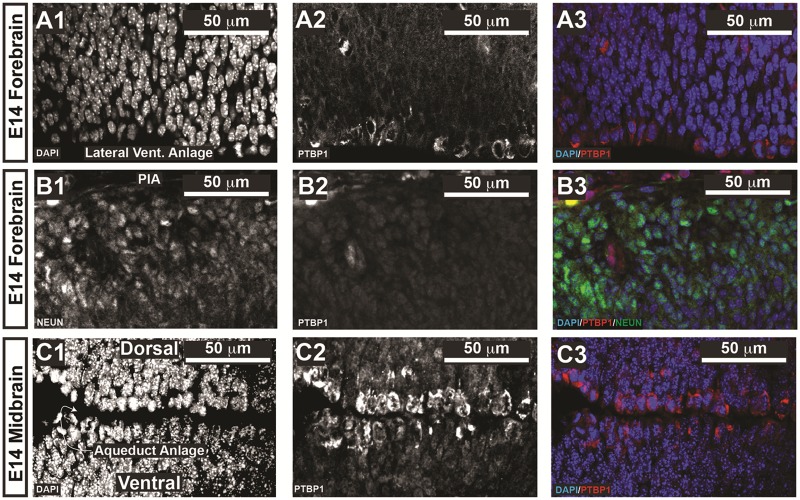
PTBP1 expression in embryonic mouse brain. Mouse embryos were harvested from timed pregnant dames and immersion fixed in 4% PFA at gestational age E14. The ventricular lining is characterized as the zone of the neural stem cell niche that undergoes cell division. In these mitotic cells, PTBP1 localized to the cytoplasm and not the condensed chromatin in the neuroepithelium lining the lateral ventricles (A) and the cerebral aqueduct anlage of the midbrain (C). In the forebrain, PTBP expression was low-to-absent in neurons (B). Color codes are paced at the bottom left of each panel.

**Fig 4:**
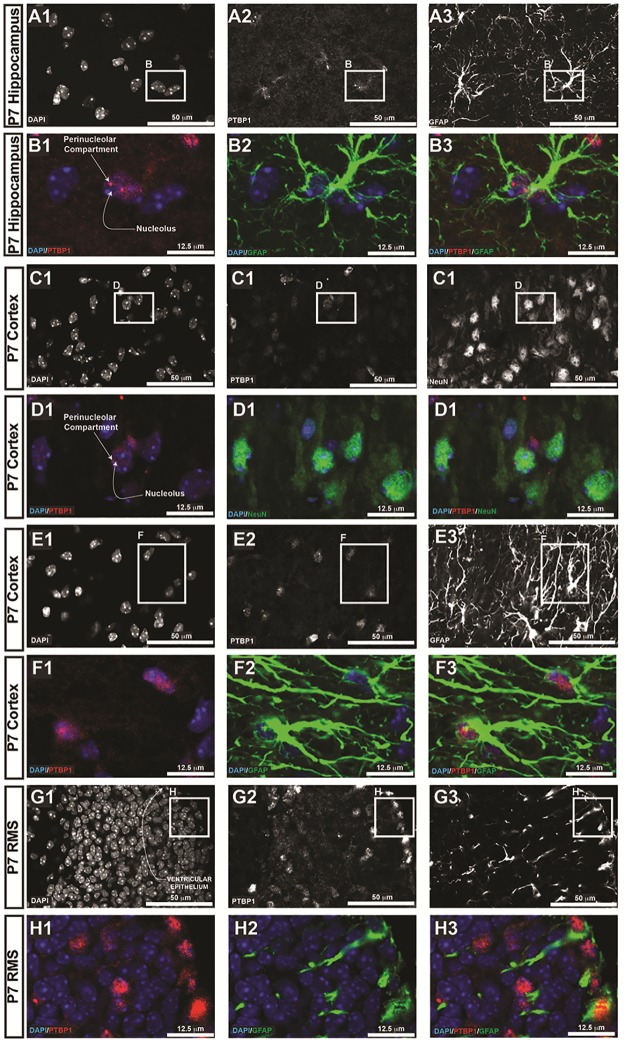
PTBP1 expression in postnatal mouse brain. Mice were perfused with 4% PFA and brains harvested for neuroanatomical analyses at postnatal day 7 (P7). Distinct anatomical regions in forebrain are denoted in boxes on the left. Hippocampal astrocytes (A-B), cerebral cortical astrocytes in layers I and II (E-F), and GFAP-positive neural progenitors in the ventricular lining (G-H) all showed strong PTBP1 expression throughout the nucleoplasm and in the perinucleolar compartment. PTBP1 expression was recued in neurons, with occasional NeuN-positive cortical pyramidal neurons showing weak PTBP1 expression in the perinucleolar compartment (C-D).

Having validated the EPR9048 anti-PTBP1 antibody, we next set out to evaluate its expression in non-neoplastic cells. Prior reports stated contradictory findings, with some authors reporting little expression in brain [9, 10, 12], whereas others have shown that PTBP expression is required in the developing brain to regulate expression levels of neuronal PTBP (nPTBP) [13]. With this in mind, we evaluated PTBP1 expression in mouse brains at different gestational ages to determine PTBP1 morphology and its distribution. We noted at E14.5, PTBP1 showed strong expression along the neural progenitors lining the lateral ventricle of the forebrain and the cerebral aqueduct anlage of the midbrain, with the remaining neural cells showing expression that was significantly weaker (Fig 3A-3C). Of note, neural progenitor cells undergo mitoses at the ventricular lining, where PTBP1 is noted to localize staining in the cytoplasm. We conclude that in embryonic neural progenitor cells undergoing mitosis, PTBP1 protein levels are increased and are not localized to chromosomal DNA. Next, we evaluated PTBP1 localization in astrocytes by co-labelling with anti-PTBP1 antibody and anti-GFAP antibodies. We noted strong PTBP1 expression in astrocytes located in the hippocampal formation and Layer I of cerebral cortex that was essentially nearly absent in neurons, although occasional neurons showed weak staining for PTBP1 in the perinucleolar compartment (Fig 4A-4H). The distribution of PTBP1 occurs in two forms: speckled throughout the nucleoplasm, whereas in other cells it shows one large punctae adjacent to the nucleolus. This perinucleolar staining is in-line with prior reports delineating a crucial role for PTBP1 in the perinucleolar compartment [11]. We conclude that astrocytes express PTBP1 in their perinucleolar compartment and throughout the nucleoplasm, and that the morphology of PTBP1 distribution is principally nuclear with a speckled pattern.

### Implementation of automated, unbiased, and quantitative image analysis of PTBP1 in diagnostic neuropathology

**Fig 5:**
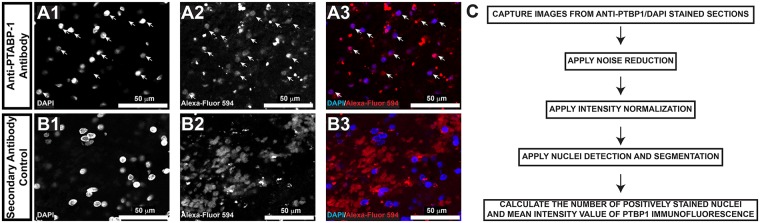
Overcoming challenges to visual interpretation of immunofluorescent images in diagnostic neuropathology. FFPE sections stained with DAPI (nuclei) and anti-PTBP1 were compared to FFPE sections stained with DAPI alone. Both were stained with secondary antibodies linked to Alexafluor-594. In (A), note that the PTBP1 signal present in nuclei (white arrows) is weaker than background autofluorescence. To underscore this, we photographed in (B) a section of the secondary control that showed significant hemorrhage. The fluorescent image coming from the red channel in (B3) represents erythrocytes. Also, note the lack of background signal in the DAPI channel (A1 and B1). (C) Image analysis workflow.

**Fig 6:**
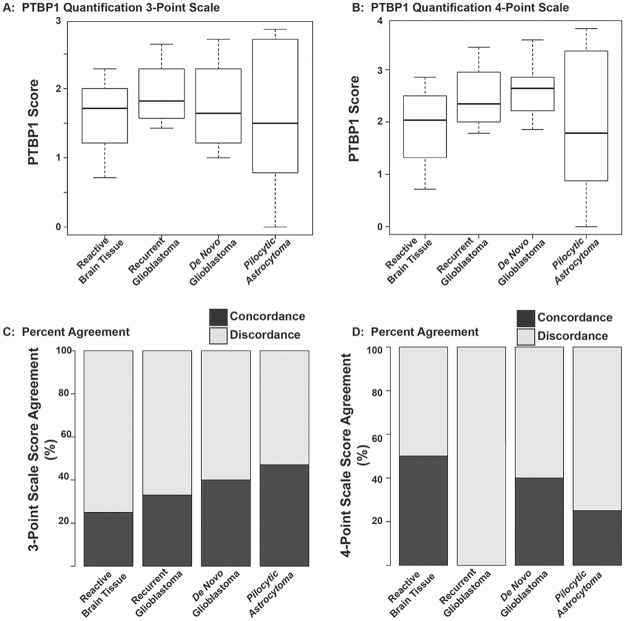
Quantification of fluorescent PTBP1 images by pathologists yields high discordance rates. (A) PTBP1 quantification on the 0–3 point scale. (B) PTBP1 quantification on the 0–4 point scale. Boxplots demonstrate median (solid bar in graph), and width of the boxes represent the first and third quartiles and thus its length demonstrates the interquartile range. The length of the whiskers are 1.5 times the interquartile range. No differences between groups was determined by ANOVA/Tukey HSD statistical hypothesis testing. (C) Discordance rate between two expert neuropathologists for the 0–3 point range. (D) Discordance rate between two expert neuropathologists on the 0–4 point range. The Chi Square Value for the 3-point scale is P = 0.86 and for the 4-point scale is P = 0.47, indicating a similar degree of discordance regardless of classification type.

Having validated our reagents and thoroughly characterized PTBP1 staining morphology, we set out to implement an automated image analysis workflow of in standard formalin-fixed, paraffin embedded tissue sections obtained from archival clinical tissue samples. We captured all data using a confocal microscope since images obtained from an epifluorescent microscope show decreased fluorescence emission samples with increasing age of the Hg bulb. We also took advantage of an idiosyncracy of FFPE prepared tissues, which is well illustrated in Fig 5. Specifically, very little background fluorescence exists in the excitation spectrum for DAPI (Fig 5A). Note that PTBP1 immunoreactivity shows localization within the nucleoplasm as a punctated morphology. However, true PTBP1 signal shows lower intensity than non-nucleoplasm associated signal. This is well illustrated in Fig 5B, which demonstrates a non-secondary antibody control with significant hemorrhage in which the fluorescent emission from the erythrocytes captured from the camera is of higher intensity than true signal (compare Fig 5B to 5A). To test the extent to which neuropathologists were able to score fluorescently labelled images of brain tissue with anti-PTBP1 antibody, we anonymized the samples and two experts (JO and AB) reviewed the material and designated a 0–3 point scale and one 0–4 points scale. Pathologists were unable to show significant differences between groups (Fig 6). Furthermore, we identified significant discordant rates between pathologists. Nevetheless, statistical analyses using squared weighted Kappa coeffecients (Table 2) showed strong agreements in the diagnostically unchallenging tumor classes of pilocytic astrocytoma and de novo glioblastoma, where both showed moderate to substantial agreement between expert neuropathologists. Note that poor inter-observer agreement was present in the diagnostically challenging categories of recurrent glioblastoma and reactive brain diagnoses. Furthermore, the 4-point analysis showed worsening concordance in each instance, underscoring the inherent deficiency for expert pathologists to subjectively evaluate expression patterns across larger categories. We conclude that without image processing, routine utilization of immunofluorescence microscopy in diagnostic neuropathology cannot be implemented.

**Table 2:** Cohen kappa coefficients for the concordance of the evaluation of pathological images.

Statistic	PA 3-point	PA 4-Point	Reactive 3-Point	Reactive 4-Point	Recurrent 3-point	Recurrent 4-point	De novo GB 3point	De novo GB 4-point
Images Scored	125	125	28	28	21	21	28	28
Raters	2	2	2	2	2	2	2	2
Kappa	0.672	0.616	0.207	0.286	0.453	0.02	0.56	0.53
Z-statistic	8.14	8.05	1.18	1.51	2.52	0.133	3.14	2.97
p-value	4.4 X10^-16^	8.8 X 10^−16^	0.237	0.13	0.0117	0.89	0.002	0.003
Interpretation	Substantial Agreement	Substantial Agreement	Fair Agreement	Fair Agreement	Moderate Agreement	Slight Agreement	Moderate Agreement	Moderate Agreement

Cohen Kappa Coefficient was calculated using a squared weighting system.

### Automated image analysis extracts meaningful data from immunofluorescent microscopic data

**Fig 7:**
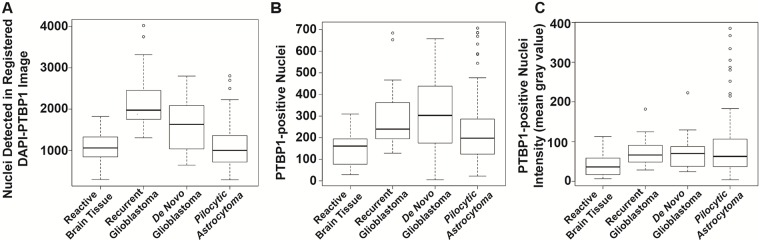
Objective image analysis demonstrates differences between groups. Objective image analysis metrics for DAPI_PTBP1 images, PTBP1-positive nuclei, and PTBP1 intensity are plotted in A-C, respectively. The median is demonstrated as the black line within the boxplot, the length of the boxplot represents the interquartile range, and the boxplot whiskers are 1.5 times the interquartile range. All datapoints outside the whiskers are considered outliers. Group to group comparisons for each analysis are performed by ANOVA and shown in Table 2.

With this in mind, we developed an image analysis workflow that allowed the automated analysis of staining that was present in the nuclei. Detailed derivation of this technique is delineated in the S1 File associated with this manuscript’s on-line material, and is briefly outline in Fig 5C. We set out to compare a series of images between reactive brain tissue versus recurrent glioma, de novo glioblastoma with recurrent glioma, and glioblastoma and pilocytic astrocytoma (PA). Since the pixel data are merged from individual DAPI and PTBP1 images for the DAPI_PTBP1 image, the detected number of positive nuclei (i.e., the pixel values in the sixth cluster) in DAPI_PTBP1 images is always higher when it is compared to the number of positive nuclei in image stained with anti-PTBP1 antibody. Therefore, numerical analyses for automated classification was based on the number of positive nuclei in DAPI_PTBP1 and in images stained with anti-PTBP1 antibody. To calculate the number of PTBP1 nuclei, we obtained the location data from the DAPI images and analyzed the brightness in the PTBP1 image. We found that with automated image analysis, recurrent glioblastoma values of DAPi_PTBP1 and PTBP1 could distinguish between these groups, whereas PTBP1 intensity was unable to identify differences between groups (descriptive statistics are plotted in Fig 7 and Table 3). We conclude that automated image analysis of PTBP1 immunofluorescence shows significant promise in being able to distinguish between reactive gliosis or recurrent gliomas in patients undergoing re-biopsy, that the expression levels (i.e., intensity) of PTBP1 are similar between recurrent glioma and de novo glioblastoma, and that the expression values between de novo glioblastoma and pilocytic astrocytoma are not significantly different.

**Table 3:** Group to group comparisons by ANOVA/Tukey HSD test.

Comparison 1	Comparison 2	DAPI_PTBP1 Intensity P value	PTBP1-positive Nuclei p valie	Intensity P value
Reactive Brain	Recurrent Glioblastoma	< 0.0001	0.0078	0.21
Reactive Brain	De Novo Glioblastoma	0.0011	0.98	0.28
Reactive Brain	Pilocytic Astrocytoma	1	.57	.007
Recurrent Glioblastoma	De Novo Glioblastoma	0.00025	0.0011	0.99
Recurrent Glioblastoma	Pilocytic Astrocytoma	< 0.0001	0.04	0.79
De Novo Glioblastoma	Pilocytic Astrocytoma	0.0000076	0.242	0.559

### The digital pathology revolution offers an opportunity to finally implement fluorescent imaging in routine solid tissue pathology

Current methods of patient selection for targeted agents rely heavily on immunohistochemical (IHC) assays. These chromogenic detection assays have several limitations in the setting of targeted therapies [14] and are subject to inter-observer variability due to subjective interpretation [15, 16]. IHC assays also permit assessment of only one pathway component at a time. Although the transition to digitized platforms in pathology has lagged radiology, pathology is now undergoing a digital revolution, with some market analyses projecting a market of over $600 million USD by 2021. Amongst the benefits of digital pathology [17] include the ability to institute quantitative and unbiased image analysis, which we previously implemented in neuropathological intraoperative cytological preparations and FFPE tissue immunohistochemical sections in brightfield microscopy [18]. However, the advent of fluorescent whole slide imaging [19] raises the possibility of implementing image analysis on fluorescently-labeled samples as we have done in this study. We propose that implementation of multiplexed fluorescent imaging in routine solid tissue pathology will ultimately permit objective quantification by automated image analysis for multiple biomarkers in each cell. We have generated an imaging analysis workflow for PTBP1 capable of extracting clinically useful information from a biomarker with a principally nuclear localization utilizing fluorescence microscopy. This technique is easily transferrable to other biomarkers showing nuclear localization, and has the added benefit of permitting multiplexed analysis of nuclear biomarkers for neuropathology. We further demonstrate that analysis of fluorescent images by pathologists is fraught with unacceptably high discordance rates which prohibits visual interpretation.

### CAP guidelines for antibody validation must include validation of antibody-epitope specificity to meet the needs of modern pathology

The CAP summary recommendations of the Principles of Analytic Validation of Immunohistochemical Assays outlines several recommendations appropriate for clinical laboratories to validate new antibodies for routine diagnostic use [2]. Recommendation 5 of these guidelines states “For a marker with both predictive and nonpredictive applications, laboratories should validate it as a predictive marker if it is used as such.” Although a great first step, we submit that this guideline is insufficient to meet the needs of modern pathology as it does not address the specificity of the antibody-antigen epitope interaction. Rather, as stated, the policy merely ensures that the immunohistochemical assays function without ensuring that the antibody-epitope interaction is valid. With these policies, antibody-epitope validation is left to antibody suppliers who as a group share no consensus guidelines for validation. This challenge brings to light several issues pertinent to modern pathology. First, insights into the molecular pathobiology of cancer have yielded new agents that target critical signaling pathways in cancer. In cancers driven by specific pathway activation, such agents have resulted in unprecedented responses and transformed patient outcome (e.g. imatinib in Bcr-Abl+ chronic myelogenous leukemia, Herceptin in Her2/Neu+ breast cancer, Ibrutinib in chronic lymphocytic leukemia). However, in cancers with more complex pathway activations, targeted agents have failed to improve outcome; in these cases, treatment resistance has been attributed to intra- and intercellular tumor heterogeneity that enable tumors to bypass single pathway inhibition. For example, *in vitro* glioma models demonstrate the presence of “bypass” signaling pathways after EGFR inhibition with activation of PI3K pathway despite erlotinib-mediated pharmacological inhibition. In this model, concurrent inhibition of PI3K re-sensitized the cells to erlotinib [20]. Thus, functional interrogation of signaling cascades are needed to identify which “bypass” pathways are activated in cancer tissues to enable selection of a tailored chemotherapy protocol optimal for each patient. Unlike Her2 immunohistochemical testing, where a backup molecular test to identify gene amplification is available [21], determination of such bypass pathways will most likely require quantification of proteins with specific post-translational modifications. Thus, to meet the need of modern oncology such biomarker evaluations will have to come from highly specific antibodies. We propose that in such instances, each antibody clone will require knock-down of the epitope of interest to demonstrate decreased antibody-epitope reactions.

### Utility of PTBP1 in diagnostic neuropathology

In this study, we have demonstrated that PTBP1 shows expression in the embryonic mouse forebrain’s neural stem cell niche as well as in postnatal astrocytes. Given the crucial role that PTBP1 plays in EGFR intracellular trafficking, we used our image analysis workflow to test the hypothesis that PTBP1 detection could aid in distinguishing between reactive gliosis/treatment affect and recurrent glioma in patients undergoing a second biopsy. We were able to find an increase in the number of PTBP1/DAPI double positive cells and an increase in PTBP1-positive cells in recurrent glioblastoma cases. We found that similar quantities of PTBP1/DAPI double positive cells and mean intensity values of PTBP1 signal were similar between de novo glioblastoma and recurrent glioblastoma, suggesting that these tissues had similar expression levels. Prior studies had suggested that PTBP1 expression was directly proportional to glioblastoma grade [10]. However, PTBP1 expression levels in pilocytic astrocytomas were not tested in this prior study. We found no statistically significant difference between pilocytic astrocytoma, WHO grade I and glioblastoma in terms of mean intensity values of PTBP1 signal. We did note an increase in PTBP1/DAPI double positive cells, but we note that the principal driver of this distinction is the well-documented increased cellular density of glioblastoma relative to pilocytic astrocytoma. Although the statistical power of this study was small, our results do point favorably for the utility of PTBP1 as a reagent in distinguishing reactive gliosis from recurrent glioblastoma.

## Supporting information

S1 Fig Image analysis of anti-PTBP images. Image stained with anti-PTBP1 antibody (a); Three homogenous regions are shown with yellow, red and cyan rectangular on the grayscale form of the image (b); Histogram of the grayscale image (c); Histogram of the regions with yellow (d), red (e) and cyan (f) rectangular. (For better visualization the brightness and contrast has been increased by 40% in Figs (a) and (b)).
(TIF)

S2 Fig Image analysis workflow. Example grayscale image that shows ROI part as yellow rectangle on the image stained with DAPI but without anti-PTBP1 antibody from PA cases (a); ROI part magnified (b); ROI part after noise reduction (c); Residual image (d); The whole image after noise reduction (e); The ROI part, its de-noised form and the residual image are shown in HSV color space in (f),(g) and (h) respectively (To increase visualization in this Fig (a-e), brightness and contrast has been increased (40%)).
(TIF)

S3 Fig Image normalization. Image stained with anti-PTBP1 antibody (a); Normalized image (b).
(TIF)

S1 File Detailed image analysis methodology. (DOCX)
