# Supplementary figures and images for: Automated fluorescent miscroscopic image analysis of PTBP1 expression in glioma
Source: PLoS One. 2017 Mar 10;12(3):e0170991. doi: 10.1371/journal.pone.0170991 (PMC5345755; doi:10.1371/journal.pone.0170991)

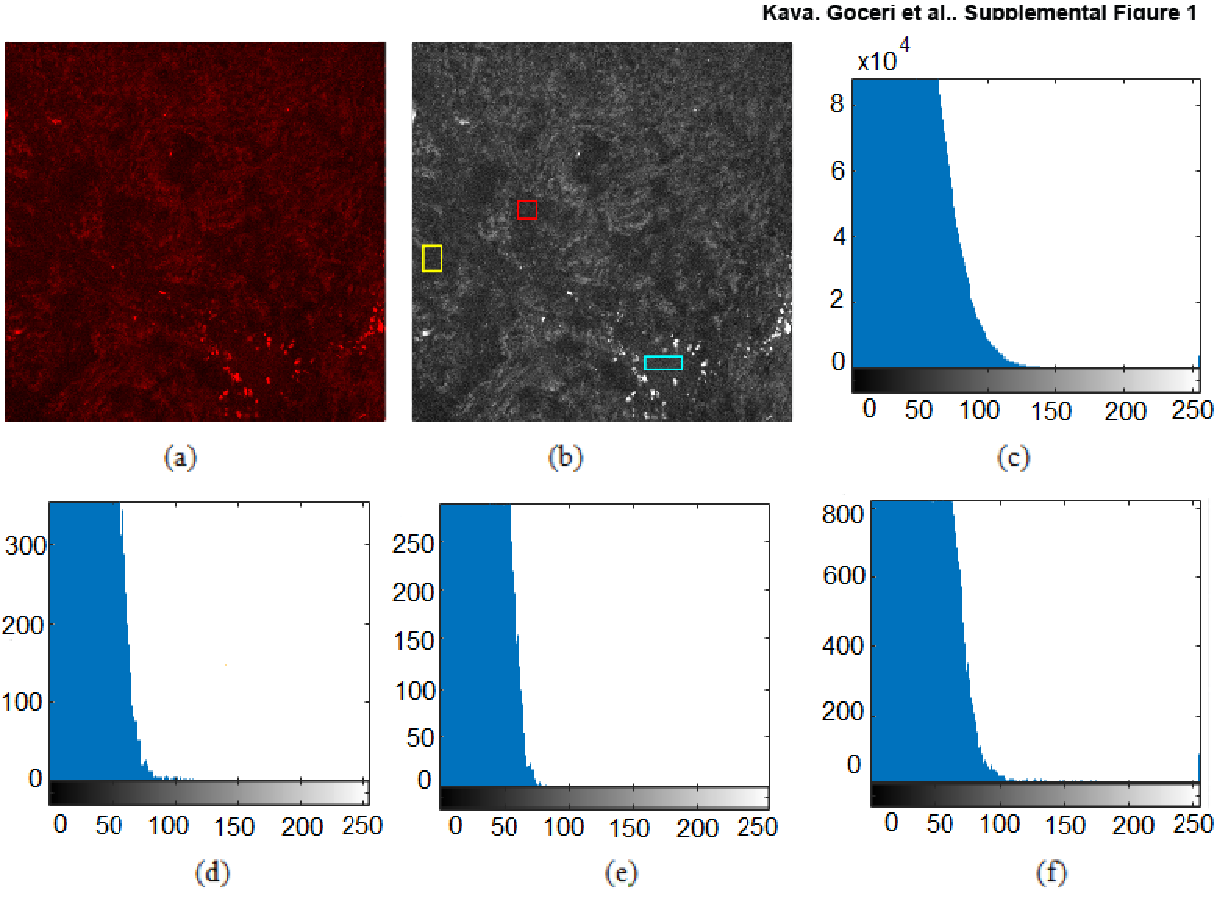

Supplement: S1 Fig — Image stained with anti-PTBP1 antibody (a); Three homogenous regions are shown with yellow, red and cyan rectangular on the grayscale form of the image (b); Histogram of the grayscale image (c); Histogram of the regions with yellow (d), red (e) and cyan (f) rectangular. (For better visualization the brightness and contrast has been increased by 40% in Figs (a) and (b)). (TIF) [file pone.0170991.s001.tif]

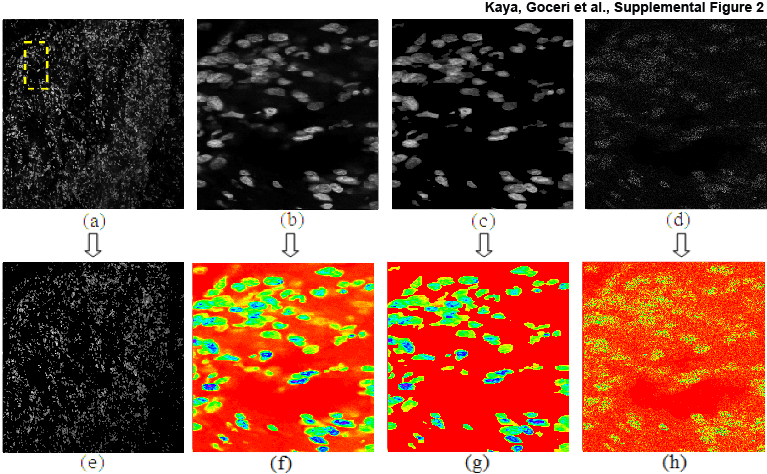

Supplement: S2 Fig — Example grayscale image that shows ROI part as yellow rectangle on the image stained with DAPI but without anti-PTBP1 antibody from PA cases (a); ROI part magnified (b); ROI part after noise reduction (c); Residual image (d); The whole image after noise reduction (e); The ROI part, its de-noised form and the residual image are shown in HSV color space in (f),(g) and (h) respectively (To increase visualization in this Fig (a-e), brightness and contrast has been increased (40%)). (TIF) [file pone.0170991.s002.tif]

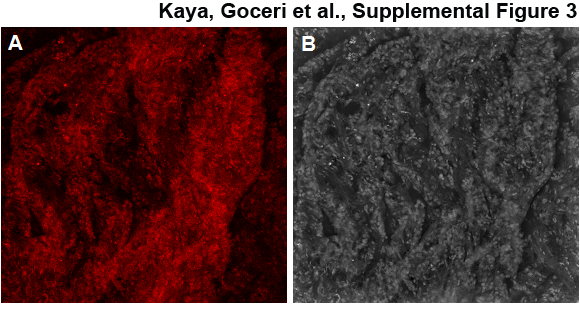

Supplement: S3 Fig — Image stained with anti-PTBP1 antibody (a); Normalized image (b). (TIF) [file pone.0170991.s003.tif]
