# Supplementary material for: Automated fluorescent miscroscopic image analysis of PTBP1 expression in glioma
Source: PLoS One. 2017 Mar 10;12(3):e0170991. doi: 10.1371/journal.pone.0170991 (PMC5345755; doi:10.1371/journal.pone.0170991)
Supplement: S1 File — (DOCX) [file pone.0170991.s004.docx]

**Supplemental Materials and Methods**

**Automated Image Analysis**

**Noise modeling and reduction**

The first step of image analysis is modeling of noise characteristics of images. The most appropriate noise reduction algorithm is implemented according to the noise model. Identification of noise type is performed with histograms of three regions (at least 100 x 100 pixels), which have homogenous or very close to homogenous intensity values, obtained from images. **S1 Figure** shows an example image stained with anti-PTBP1 antibody (**S1A Figure**), three regions (with $\sigma_{1}=0.5$, $\sigma_{2}=2.5$ and $\sigma_{3}=1$ variances), which are surrounded with yellow, red and cyan rectangular, on the grayscale form of the image (**S1B Figure**) and the histogram of the grayscale image (**S1B Figure**). We have modeled the noise in the images as speckle noise based on the shape of the histograms (**S1D,E, F Figure**) of those three regions. Therefore, we applied a Non-local Median based Speckle Filtering (NMSF) (Goceri, et al, submitted) for de-noising.

The effect of the applied de-noising method is illustrated with a Region of Interest (ROI) area, which is shown with the yellow rectangle in **S2A Figure**. (The ROI part is magnified in **S2B Figure** to increase visibility of individual nuclei). The ROI part after noise reduction (**S2C Figure**), residual image (i.e. difference between the original and de-noised ROI area) (**S2D Figure**) and the whole image after noise reduction (**S2E Figure**) are shown as grayscale. To present better visualization, pseudo-color representation with the Hue-Saturation-Value (HSV) color model is given in **S2F,G,H Figure** for the images shown in **S2B, C, D Figure** respectively.

**Local Intensity Normalization**

The second step is intensity normalization to tackle the problem of non-uniform staining in images. The local normalization method applied in this step is based on Gaussian filtering with adaptive sigma (Goceri, et al., submitted). An example result obtained by the normalization method is given in **S3B Figure** with an image stained with anti-PTBP1 antibody (**S3A Figure**).

**Nuclei Detection and Segmentation**

The third step is detection and segmentation of cell nuclei stained positively with anti-PTBP1 antibody and DAPI. A deterministic technique (Goceri, et al., submitted) is applied in this step. This technique is based on brightness in images since positively stained nuclei in images with anti-PTBP1 antibody appear brighter than other objects. Therefore, an image, which we called as *DAPI_PTBP1*, showing shapes in images stained with DAPI and textures in images stained with anti-PTBP1 antibody was generated. The DAPI_PTBP1 image is clustered with a k-means algorithm [Wu, J. Advances in K-means Clustering. Springer Theses Springer; Berlin Heidelberg, Cluster Analysis and K-means Clustering: An Introduction.; p. 1-16. DOI: 10.1007/978-3-642-29807-3, 2012 ] into six clusters (i.e., k=6), which indicate 1)black (background), 2)dark gray, 3) gray, 4) bright, 5) brighter and 6) the brightest gray level value. The sixth cluster shows the nuclei positively stained with anti-PTBP1 antibody. **Figure 5C** in the main text shows the flow chart applied in this section.
